# Development of an Internet-delivered educational video for acute whiplash injuries

**DOI:** 10.1186/s40814-019-0445-2

**Published:** 2019-04-27

**Authors:** Majbritt Mostrup Pedersen, Per Fink, Helge Kasch, Lisbeth Frostholm

**Affiliations:** 1 The Research Clinic for Functional Disorders and Psychosomatics, Aarhus University Hospital, Nørrebrogade 44, Bygn. 2C, 8000 Aarhus, Denmark; 2 The Spinal Cord Injury Centre of Western Denmark, Department of Neurology, Regional Hospital of Viborg, 8800 Viborg, Denmark; 3 Department of Clinical Medicine, Aarhus University, 8000 Aarhus, Denmark

**Keywords:** Whiplash injuries, Patient education, Patient information, Illness beliefs, Illness perceptions

## Abstract

**Objective:**

To describe the development of a preventive educational video for patients exposed to whiplash trauma following motor vehicle accidents.

**Methods:**

The development followed a systematic approach and was theory-driven supplemented with available empirical knowledge. The specific content was developed by a multidisciplinary group involving health professionals and visual production specialists.

**Results:**

A 14-min educational video was created. The video content focuses on stimulating adaptive recovery expectations and preventing maladaptive illness beliefs. The video presents a multifactorial model for pain incorporating physiological and cognitive-behavioural aspects, advice on pain relief, and exercises. Subjects interviewed for a qualitative evaluation found the video reassuring and that it aligned well with verbal information received in the hospital.

**Conclusions:**

The development of the visual educational intervention benefitted from a systematic development approach entailing both theoretical and research-based knowledge. The sparse evidence on educational information for acute whiplash trauma posed a challenge for creating content. Further knowledge is required regarding what assists recovery in the early stages of whiplash injuries in order to improve the development of educational interventions.

## Background

Whiplash injuries following motor vehicle collisions affect an estimated 1–3 per 1000 inhabitants each year [1]. The movement of the neck and head during impact can result in varying degrees of injuries but is most commonly associated with benign soft tissue injuries to the neck [2–4]. An estimated 25% develops severe persistent pain affecting long-term work ability, daily function, and quality of life [5]. Delayed recovery is not satisfactorily explained by detectable patho-anatomical changes [6–9]. Instead, a plethora of complex and intertwined characteristics such as initial pain levels, reduced cervical range of motion, PTSD symptoms, age, gender, education, depression, and pre-injury sick leave have been associated with negative outcomes [10–15].

Currently, no specific treatment is available for standard care due to the lack of consistent evidence for the effectiveness of any treatment strategy [16, 17]. Management in the acute phase thus primarily consists of efforts to detect and exclude the presence of serious injuries such as fractures. If no such injury is found, guidelines recommend providing reassurance and some form of patient education [1, 4].

The recommendations for providing patient education reflect evidence that early mobilization and advice to act as usual may improve recovery to some extent [18, 19]. Furthermore, more intensive early treatment efforts have so far not yielded convincing effects above what can be achieved through simple advice [20–23]. Noteworthy, there is even evidence that early intensive treatment efforts may worsen outcome [24–27]. It has been suggested that these negative effects may stem from health professionals unintentionally promoting maladaptive beliefs about the seriousness of the injury when providing extensive treatment [25, 26].

Several studies have found an association between early pessimistic illness beliefs and expectations and poor recovery rates [28–34]. These findings indicate that targeting beliefs concerning the nature and course of the injury might be important. Given that illness beliefs may be modifiable in the early phases of illness [35], early patient education targeting beliefs and expectations about the injury could have a pivotal role.

The verbal information provided in the emergency room immediately after the accident is often the only patient education affected individuals receive [36]. The time available for emergency room staff to engage with patients is limited on busy shifts, and the patient may be in pain, feel exhausted, and emotionally affected by the accident further complicating the delivery of patient education. Contextual factors thus present a challenge for knowledge transfer and retention. In light of these challenges, there is a need for the development of less time-consuming and more easily administered patient education for the acute phase.

Providing patient education online in a visual form could be a pragmatic expansion of the face-to-face information in the emergency room. Video education is equal to face-to-face information when it comes to improving patient knowledge and may also promote behavioural changes and improved self-care [37]. Visually based information may be considered less demanding than written material [38, 39] and thus more appropriate for acutely injured patients. Furthermore, visual presentation allows for demonstration of complex behaviour through modelling where another patient performs the recommended behaviours. This strategy may be superior to just providing factual information [39, 40].

So far, only two studies have attempted using patient education by video for whiplash injuries [41, 42]. A Canadian trial provided a DVD at home a few days after the accident and found a trend towards better improvement in the intervention group in comparison with controls [41]. A smaller American study showed a video in the emergency room before discharge and found a significant improvement in pain report and use of analgesics 6 months later [42].

On the basis of the aforementioned studies, we aimed to develop a novel educational video for individuals presenting in Danish emergency rooms with pain complaints after whiplash trauma. The goal was to expand the verbal information that is provided as standard care with information specifically aimed at promoting adaptive beliefs about the nature and the course of the injury. The development of the intervention took place during the preparatory phase for a randomized controlled trial aimed at testing the effectiveness of an educational video on subsequent pain levels, disability, and work ability following acute whiplash trauma.

## Methods

### Development strategy and target population

**Fig. 1:**
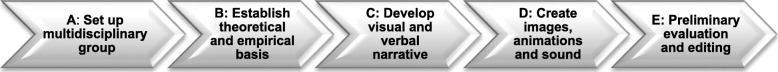
Stages in the development of the intervention

Interventions are often developed in an ad hoc fashion without an extensive theoretical or empirical foundation [43]. This may in part stem from the fact that many interventions are developed in the context of a pragmatic clinical treatment effort. However, as underlined by the Medical Research Council [43], the development of any health-related intervention should be firmly informed by existing theoretical and empirical knowledge. It has been pointed out that the reliance on pragmatism may be especially pronounced when it comes to the increasing use of visual media in the health sector [44]. Williams and colleagues [44] therefore created an elaborated model for the development of health-related visual media adhering to the general recommendations by the Medical Research Council. The model was adapted to the specific challenges and requirements of visual media in order to function as a guide for the development process. We used that model as an inspiration for implementing a step-wise approach to developing the video (see Fig. 1). The video content was developed in 2012.

The target population for the educational video was specified as persons exposed to a likely whiplash trauma during a motor vehicle accident and seeking care in an emergency room due to acute neck complaints. Fractures, concussions, or any other direct trauma or injury were excluded. The intended recipients thus correspond to whiplash-associated disorder (WAD), grades 1–3 as specified in the Quebec Task Force classification [4]. The intervention was intended for use after a qualified clinician had verified that no serious injury was present and within 72 h of the accident. The recipients had to be fluent in Danish and aged 18 or above. The minimum age requirement was found appropriate as current knowledge on whiplash injuries originates from studies including adults only, and it is uncertain whether evidence from studies of adults can be applied to children.

### Setting up a multidisciplinary development group

A multidisciplinary group responsible for the development and the content of the intervention was established. The group consisted of a neurologist with expertise on whiplash and pain treatment, a psychiatrist specializing in functional somatic syndromes, a psychologist with expertise on illness perception and cognitive behavioural therapies, a photographer with extensive experience in video production for patient information within the hospital-based healthcare system, and an experienced animator. A second psychologist familiar with pain treatment acted as the coordinator for the group and was responsible for writing the draft for the intervention. Other health care professionals were consulted for advice during the different stages of the development process. Amongst these were a consultant from an emergency department, a physiotherapist, a physician specializing in the treatment of chronic bodily symptoms, and a psychologist specializing in whiplash.

### Establishing a theoretical and empirical basis—the creation of conceptual content

The multidisciplinary group focused on one key theoretical concept as a theoretical framework for the development of the visual material, namely the common sense model of illness developed by Leventhal and colleagues [45]. The model proposes that patients build mental models of their illness shaping the behavioural and emotional response to the condition. These mental models are often referred to as illness perceptions (IP) [35, 46]. According to the model, IP interact with symptoms and coping strategies thereby exerting important influence on the course of a given health threat. It has been shown that IP influence several health-related outcomes such as utilization of health care, self-management behaviours, quality of life, and disability across a range of different diseases [46–49]. IP can be divided into five components: identity, causal beliefs, timeline beliefs, control beliefs, and consequences (see Table 1).

**Table 1:** Defining components of illness perceptions (IP)

Illness perceptions (IP)	
Identity	The name and the symptoms the patient ascribe to the condition
Cause	The perceived cause of the condition
Timeline	The expected duration of the symptoms
Control	The appraisal of whether the condition is controllable or not
Consequences	The expected impact of the condition on the subjects’ life

By addressing the five components of IP, we aimed at increasing the patients’ ability to cope appropriately with acute symptoms and thereby preventing the transition to prolonged pain. Furthermore, the intention was to counteract misconceptions and possible myths surrounding whiplash injuries [50–52]. Examples of how the theoretical concept of illness perceptions (IP) guided video content are provided in Table 2.

**Table 2:** Example of the theoretical concept of illness perceptions (IP) guiding video content

Dimension of illness perceptions (IP)	Related video content
Identity	Providing an appropriate name (sprain), explaining the correct origins of the term whiplash to avoid misconceptions, providing overview of typical symptoms.
Causal beliefs	Attributing symptoms to minor and reversible soft tissue injuries comparable with a sprain in other body parts. Prolonged symptoms connected to central sensitization/imbalance in the regulation of the pain system (disconnecting pain from tissue damage).
Timeline beliefs	Providing a typical time frame for healing of a soft tissue injury or sprain of 6–8 weeks.
Control beliefs	Providing information about what the patient can do to assist the healing process and manage pain and discomfort.
Consequences	Information about the generally good prognosis. Emphasizing that it is safe to gradually resume daily activities in spite of varying degrees of pain.

While the concept of IP provided an overall frame and goals for the intervention, the information specific to whiplash injuries was extracted from empirical research. An earlier review of available scientific evidence concerning prognosis and management of whiplash-related injuries [53] was used as a starting point for selecting information to be addressed in the video. A patient education booklet developed by Waddell and colleagues in connection with the review [54, 55] was discussed in the multidisciplinary group. Peer-reviewed papers published after the aforementioned review [53] were then searched to establish whether new relevant knowledge had come to light.

Medline and Cochrane Database of Systematic Reviews were searched for papers published in the preceding 10 years (2001–2011) containing the term “whiplash injuries”. All titles and abstracts were examined to evaluate relevance. Especially, studies related to patient expectations and beliefs were considered due to the chosen emphasis on promoting optimistic IP. Fear of movement and self-efficacy [56–62] were therefore included as central concepts to target in the intervention. In light of research findings indicating that immobilization is associated with reduced recovery rates, we chose to put an emphasis on preventing passive or avoidant responses to pain. Promoting early mobilization and an active approach to recovery in the patients would hypothetically correspond to reducing fear of movement and enhancing self-efficacy.

Two studies on video education aimed at acute whiplash trauma [41, 42] were considered to contain essential material. Permission was therefore obtained from authors to replicate and remake relevant information from the two videos for the Danish video. Since both videos were created in a different cultural setting (i.e. the USA and Canada) using a foreign language and explanatory models for symptoms that are not routinely used in a Danish medical setting, it was not found suitable to reuse the videos in Denmark. In order for the video to function as the intended extension of standard care, it was a priority to ensure a high degree of coherence between information received in the emergency room and the video content. The development group reached consensus on a written description of the central concepts and information to be included in the video. This provided the basis for creating a manuscript for the video production.

### Creating a visual and verbal narrative

The next step was setting a structure and shaping the visual and verbal narrative of the video. A time frame of 10–15 min in length was set for the video. This was deemed a proper compromise between having enough time to provide adequate information without overtaxing the attention span of patients with pain and possibly difficulties sustaining attention. Discussions were focused on deciding what specific information to represent and finding the balance between different types of information (general information, behavioural information, and possible exercises). The group developed ideas for the visual representations needed to convey the intended message and discussed the overall balance between suggested animations and footage of patients and professionals. On the basis of research on patient education through video [39, 40] suggesting that modelling of desired behaviour by a realistic model is the most effective way of reassuring patients and creating behavioural changes, it was decided to emphasize footage of model patients.

Selected elements from the aforementioned American and Canadian videos [41, 42] were adapted and reworked to suit the Danish version. The included physical exercises were reproduced from the American video as these were considered relevant and safe for acute patients. The exercises were of such a gentle nature that no adverse effects would be expected, even if they were not carried out in the prescribed way. They were therefore found safe to use without face-to-face instruction from a practitioner. Even though specific neck exercises have been shown to aid recovery in some studies [63–65], the purpose of including exercises was primarily to counteract the fear of movement or reluctance to move the neck and resume daily activities due to pain. As well as providing a visual demonstration showing that it is harmless to move in spite of pain, it was hypothesized that providing exercises and advice on the resumption of daily activities could promote self-efficacy. Rather than exerting a direct physiological influence on symptoms, the exercises were intended as a vehicle for promoting a general early mobilization of the neck. Finally, considerable time was dedicated to discussing the phrasing of the verbal information in order to make the video readily understandable for laypersons with varying educational background.

The work within the multidisciplinary group resulted in a detailed manuscript for a video with an estimated length of 15 min. The manuscript contained both the exact phrasing of the verbal content and a description of the visual representation including the different scenarios and persons that should be featured as well as suggested animations, graphs, and exercises. The detailed manuscript was presented to a medical consultant and a physiotherapist with extensive experience with patients suffering persistent symptoms after whiplash traumas. The responses to the manuscript were considered by the multidisciplinary group, and final adjustments were made to the manuscript. The comments were primarily concerned with adjustments to the phrasing of the verbal content, and while achieving complete agreement on all comments was not possible, a satisfactory compromise was reached.

### Creating images, animations, and sound

The manuscript was reworked by the coordinator, photographer, and animator from the Department of Communication at Aarhus University Hospital. The visual structure of each segment of the video, including a detailed plan for visual scenes, flow of the images, and the appearance of animations and graphics, was made in a manner equivalent to creating a storyboard. The final manuscript was then reviewed and approved by the multidisciplinary group.

**Fig. 2:**
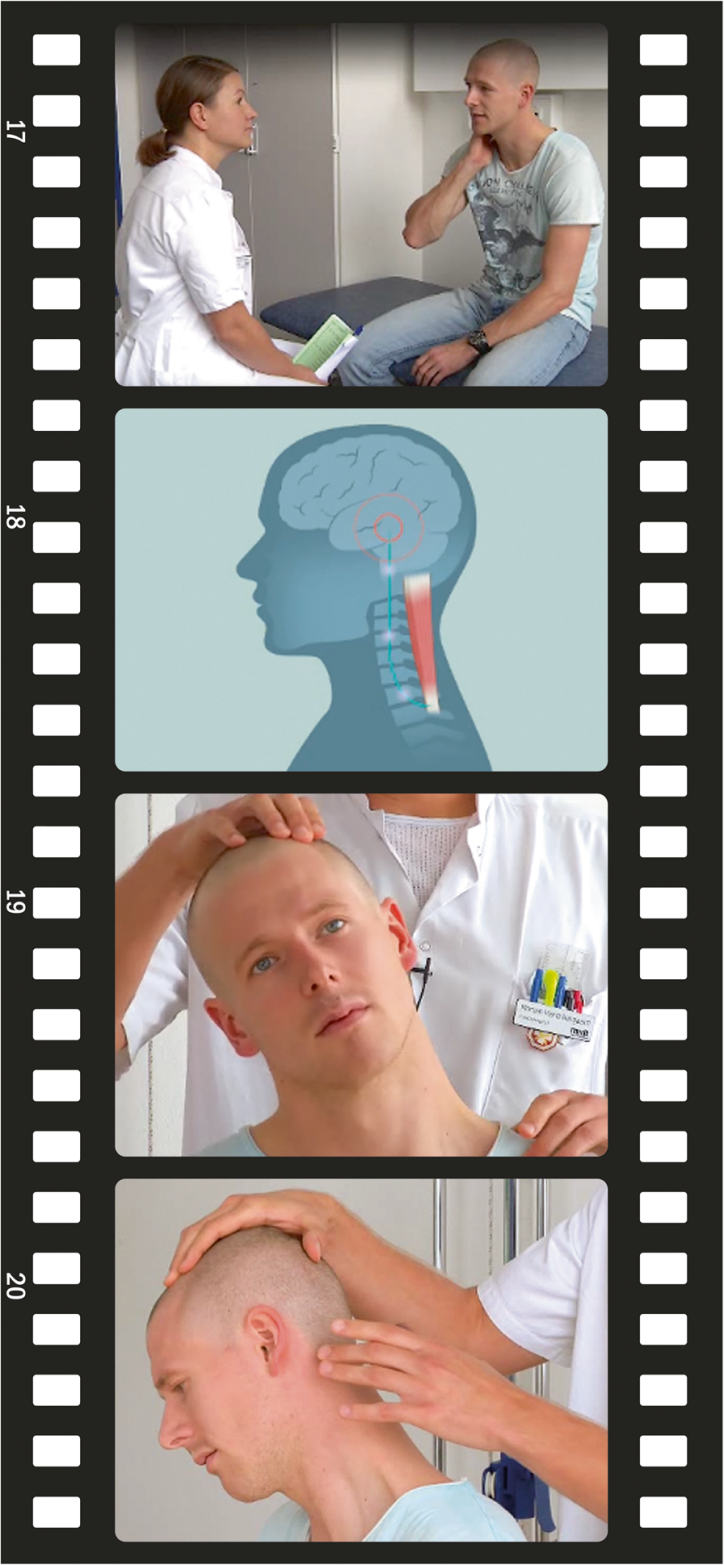
Images of filmed video segments

The video sequences were filmed over a period of 2 months at different locations with simultaneous development of the animations. We used a combination of filmed sequences with doctors and patients coupled with animations and graphs (see Fig. 2). These were supported by verbal explanations provided by a male voice-over. A professor of neurology and a neurologist specializing in pain and whiplash featured as medical experts, while a hospital-based physiotherapist presented the mobilizing exercises. A psychiatrist with extended qualifications in mindfulness techniques presented a relaxation type exercise. Two actors (one male and one female) featured as patients in the video and modelled the basic advice on pain relief, gradual return to daily activities, and performed the specified exercises.

### Preliminary evaluation and final editing

The process resulted in an approx. 17-min-long version of the video. The pre-final edit was shown to a diverse group of persons who had not previously seen any of the video content. Among these were a psychiatrist, doctors, and nurses working in an emergency room; an experienced journalist working with TV production; and six laypersons with differing educational background, two of whom had previously experienced a whiplash trauma themselves.

Professionals were more critical of the content than laypersons (see Table 3). The feedback from the viewings was discussed by the multidisciplinary group, and the visual production personnel attempted to include critique given in the final edit of the video. This resulted in changes to the tone of the speech and inclusion of slightly more footage of the patient modelling desired behaviour while reducing footage of doctors and hospital settings. Adjustments were also made to sound in terms of removing audible background noise and to colour schemes, and the overall length of the video was reduced. The final editing process was however limited by the lack of options to record new material. This was primarily due to time and budget constraints. Recording new material at this stage would have proven costly and time consuming because settings, seasons, and participants had changed visually or were unavailable for filming. In order to retain the visual coherence of the video and avoid unnecessary visual distractions from the intended message, the editing process was therefore restricted to existing footage. A compromise that was found acceptable by the multidisciplinary group was reached.

**Table 3:** Focus points in evaluation feedback from professionals and laypersons

Professionals focused on	Laypersons focused on
• That laypersons would not understand specific phrases used (no consensus between different professionals on exact phrases) • Details of animations and visuals (often suggesting more detail or expansions to explain symptoms) • Found visual content calming • Suggested improvements for making the visual edit appear more professional • Improvement of sound and lighting • Questioning whether participants were convincing and skilled in presentation and looking into the camera	• Whether the doctors seemed nice and which doctor they personally preferred to listen to (differed greatly) • Found the footage and animations convincing and credible • Found information easily comprehensible • Content acceptable and calming • The tone of speech was too low/dark • Speech not clearly audible in all segments • The speed of the edit was found appropriate • Preferring footage of “what to do” rather than doctors giving too much detailed information • Would prefer a slightly shorter video

## Results

### The resulting educational video

The development process resulted in a 14-min-long educational video aimed at patients experiencing symptoms after an acute whiplash trauma. An overview of the themes and content of the video can be found in Table 4. The video is designed for viewing within the first 72 h following the accident and shaped to refresh and expand the reassurance and advice that the patients receive from the attending physician in the emergency room.

**Table 4:** Theme-based content of educational video

Theme (segment)	Content	Purpose	Visuals and sound
Introduction	Short introduction to the video and the purpose.	Understanding the purpose of the video.	Consultant speaking to the viewer in a hospital setting.
“Injury model”/biopsychosocial model for whiplash trauma	Explaining “whiplash” mechanism of trauma and the expected acute effects on soft tissues.	Providing a clear understanding of what a whiplash injury is. Preventing “myths”.	Animation of rear-end-collision and head-neck-movement. Focus on soft tissues.
Expectations about symptoms	Description of common symptoms that the patient may experience in the acute phase.	Providing expectations and reassurance concerning symptoms.	Consultant speaking and showing on patient which areas of the neck are involved.
Biopsychosocial model as framework for understanding symptoms	Simple biological model for acute pain focusing on muscle tensions and soreness.	Establishing connection between symptoms and soft tissues with a high capacity for healing.	Animation of muscle tension and soreness. Verbal explanation by professional speaker.
Prognosis and expectations about the future	Description of prognosis, healing time, and pain in acute over sub-acute phase.	Establishing realistic expectations about recovery and preventing excessive worry.	Consultant coupled with animation of expected typical healing time and expected pain.
Gradual mobilization and self-efficacy/empowerment	Description of the importance of early gradual mobilization of the neck.	Establishing confidence in moving the neck and being active in promoting recovery.	Recording of consultant coupled with patient moving head and neck.
Acute pain relief	Information about basic pain relief in the acute phase. Pain medication, use of short-term rest, use of cold/hot packages.	Promoting active self-care behaviour and demonstrating how to use basic pain relief strategies.	Recording of patients illustrating the use of techniques for pain relief.
Biopsychosocial model: understanding sub-acute symptoms.	Factors that can cause symptoms to fluctuate in the healing process: physical strain, lack of adequate movement and emotional and social strain	Understanding the fluctuating nature of symptoms in the sub-acute phase and how symptoms might be aggravated.	Revisiting animations of muscle tensions and patient moving. Examples of factors that can aggravate symptoms (i.e. stress, lack of movement).
Sub-acute management of symptoms	Information about gradual return to daily activities and normalization of discomfort	Promoting movement and attention to initial stages of pain before pain escalates.	Patients in daily life situations. Verbal explanations provided by professional speaker.
Sub-acute management of symptoms: exercises	Appropriate stretch and relaxation exercises for relief of pain and muscle tension.	Providing pain relief exercises, reassuring that movement is appropriate	Physiotherapist instructs patient in stretch exercises, doctor performs relaxation exercise.
Summary of central messages	Short statement about positive prognosis and the patient’s active role in promoting recovery through gradual return to daily activities.	Providing a reassuring and unambiguous take-home message promoting positive expectations and focus on return to daily activities.	Recording of consultant briefly underlining central points with bullet points appearing one by one on the screen.

The content of the video aims to demystify the acute condition and establish realistic expectations for prognosis and the course of experienced symptoms. It communicates a readily understandable biological model for the presumed injury which is likened to a sprain of the neck. A presumed causal association between benign and self-limiting soft tissue injuries and commonly experienced symptoms is thus presented and coupled with optimistic expectations for recovery. The explanatory model also involves simplified explanations of factors that can result in persistent symptoms in spite of normal tissue healing. Specifically, the information touches upon prolonged muscle tension, overdoing or underdoing physical activity, stress, and dysregulation within the pain system itself. The video advises to mobilize the neck and return gradually to everyday activities while also providing exercises as simple means of coping with acute symptoms.

### Postproduction qualitative evaluation

Semi-structured telephone interviews were conducted in order to provide a preliminary qualitative evaluation of the intervention. Four patients (two females and two males) receiving the video after their visit to the emergency room were interviewed and gave account of their experience of the information.

All the patients felt the video was acceptable and that it had been a positive experience to receive the intervention. Likewise, they all felt the content was relevant, helpful, and reassuring to watch. They also noted that the content was readily understandable and corresponded well to the information they were given by staff in the emergency room. All four preferred to receive a video instead of written material.

Two patients explained that it was helpful to watch the information at home as they were distraught and in pain when they were in the emergency room. They therefore found it difficult to focus on the information provided by the staff. Only one out of four had used the exercises presented, and this one patient found them helpful and attributed her ability to use them to the visual presentation. One patient noted that he did not actively use any of the information in the video but felt that it had a calming effect on him and made him feel that the hospital staff took his pain seriously. Another patient noted that he had difficulties assessing how much pain he should tolerate during movement and would have liked more specific information about this.

## Discussion

This paper describes the process of developing a brief educational video for acute whiplash injuries. In many cases, new interventions are not developed in a systematic transparent manner or described in detail making it a challenge to interpret and replicate studies. It is a clear strength of the current study that we used a structured model for the development of the intervention. Furthermore, it is an advantage that we involved a multidisciplinary team and personnel with expertise in visual media from the very beginning as recommended by Williams and colleagues [44]. In addition, we used a theory-driven scaffolding in the form of IP for establishing the primary goals and directions of the intervention and building the content. Finally, we made extensive efforts to include content that reflected available evidence from scientific studies concerning acute whiplash injuries.

While the systematic approach to developing visual interventions is recommendable, it is also time-consuming and requires the involvement of personnel from different disciplines, all of which can pose a challenge in a busy hospital setting. It is a possible weakness of this study that the patients’ perspective was not included to a higher degree at the very inception of the video. For future projects, it would be recommendable to take a more patient participatory approach. This could be achieved through a qualitative examination of patients’ wishes for information before filming is initiated or by including patients as members of the multidisciplinary development group. Doing so would in all likelihood ensure the best possible match between patients’ needs and the final product. Ideally, we should also have included an extensive qualitative testing of the video segments early in the development process before reaching a final edit. This was originally proposed by Williams and colleagues [44], but we did not opt for this due to time pressure. We would however recommend to pilot test visual interventions with relevant patients at a very early stage of the development. This may not only help establish which content is the most helpful and if anything is lacking but also aid the shaping of visual form and verbal phrasing. It may also be considered a weakness that none of the theoretical concepts utilized in the video have been specifically developed for the injury in question, and it is currently unclear to what extent these concepts can be transferred to whiplash. For example, the concept of fear of movement, which may be considered relevant in low back pain [66], has so far yielded variable results in studies related to whiplash [58, 67–70].

The video was specifically tailored to provide optimistic expectations concerning recovery. However, it is a point for discussion whether or not one ought to include information about the possibility for experiencing prolonged symptoms. As pointed out by Rebbeck [71], guidelines on management of acute whiplash opt for an optimistic and reassuring strategy, but in fact, we know very little about the possible effects of providing early optimistic expectations that may not be fulfilled for everyone. This argument is partly based on a qualitative study which found indication that it can cause worry when symptoms do not resolve in the manner that the patient had initially been assured of [72]. During development of the video, it was discussed whether to include information on prolonged symptoms, but we ultimately chose not to due to the focus on promoting optimistic IP. Instead, we opted for including general information on when to seek guidance from health practitioners in case of aggravated or prolonged symptoms. This may still be the preferable option when designing a “one-size-fits-all” intervention for acute whiplash, where the majority of patients only experience self-limiting and transient symptoms.

The major challenges we faced during the development of the video were not related to the systematic approach itself but rather reflected the state of the available empirical evidence. The sparse number of prior interventions, which have proven helpful in aiding recovery, meant that the choice on video content relied heavily on knowledge on prognostic factors as opposed to content with an established impact on recovery. Another challenge we faced while reviewing the scientific literature as guidance to the information was the broad range of risk factors associated with persistent pain after whiplash trauma [11, 73]. Several of these could be addressed, and it is unclear which risk factors would be most beneficial to target. This leaves a risk that we have included information with little impact on recovery while not expanding on concepts that might hold a larger potential for promoting recovery.

Further complicating the matter, studies suggest that patients affected by whiplash trauma are a heterogeneous group involving subgroups with significant differences in clinical outcome [5, 33]. Creating an intervention that is helpful for the entire range of patients while adequately targeting risk factors that may only be relevant for subgroups is challenging. It is possible that tailoring educational efforts in the acute stage to specific subgroups would be a way forward. Furthermore, it would be helpful for the development of future educational interventions to perform qualitative studies on patients’ perceptions of coping with the injury in the early stages. This could indicate what information the patients would likely benefit from in the acute phase. It remains to be seen whether this would correspond with information from studies of chronic patients and prognostic factors.

Aside from the challenges of tailoring evidence-based content, video can be a practical tool for standardized patient education that is easy to implement once created. Since time has moved beyond reliance on physical devices such as DVDs, creating an Internet-delivered solution is in our view the most pragmatic strategy for implementation. Due to the widespread use of electronic devices with access to the Internet in the general population, providing access online will make the intervention relatively cheap to disseminate. It means patients can watch the information in the waiting area of the hospital, on the commute home, at home, or at work at their own discretion. We therefore believe that Internet-based videos will be an important tool for disseminating health-related information in the future.

## Conclusion

An educational video for acute whiplash injuries was developed by a systematic approach. The model guiding the development process proved helpful in combining theoretical considerations, e.g. illness beliefs, with available empirical knowledge on whiplash injuries. Furthermore, the approach ensures that extensive consideration is given to the development of conceptual and visual content without compromising the importance of incorporating both theoretical and evidence-based knowledge. The strategy can easily be adapted to other patient groups and settings provided that the required personnel and technical resources are available.

The primary obstacles in the development process were not the requirements of the systematic approach but the relatively sparse scientific evidence on information expected to promote recovery after whiplash trauma. More extensive knowledge is required regarding what assists or hampers recovery in the early phase after injury in order to guide the information presented to patients.

The effectiveness of the educational video presented here is currently being tested in a randomized controlled trial (Clinicaltrials.gov ID: NCT01699334).

## Abbreviations

IP: Illness perceptions
WAD: Whiplash-associated disorder
